# Healthcare Waste Toxicity: From Human Exposure to Toxic Mechanisms and Management Strategies

**DOI:** 10.3390/jox15050155

**Published:** 2025-09-25

**Authors:** Ilie Cirstea, Andrei-Flavius Radu, Ada Radu, Delia Mirela Tit, Gabriela S. Bungau

**Affiliations:** 1 Doctoral School of Biological and Biomedical Sciences, University of Oradea, 410087 Oradea, Romania; cirstea.ilie@student.uoradea.ro (I.C.); dtit@uoradea.ro (D.M.T.); gbungau@uoradea.ro (G.S.B.); 2 Department of Psycho-Neuroscience and Recovery, Faculty of Medicine and Pharmacy, University of Oradea, 410073 Oradea, Romania; 3 Department of Pharmacy, Faculty of Medicine and Pharmacy, University of Oradea, 410028 Oradea, Romania

**Keywords:** healthcare waste, waste toxicity, toxic mechanisms, waste management, chemical wastes, hazardous waste

## Abstract

Healthcare waste (HCW) represents a growing yet frequently underestimated threat to public health, due to its complex toxicological profile. Exposure to HCW has been associated with a broad spectrum of adverse effects, including infections of bacterial, viral, or fungal origin, as well as systemic consequences such as endocrine disruption, metabolic disturbances, and mutagenic, carcinogenic, or teratogenic outcomes. These risks are particularly elevated among healthcare professionals and waste management personnel, who are directly exposed to hazardous materials. This narrative review aims to consolidate current knowledge on the toxic potential of HCW, emphasizing the variability of risks according to waste category and point of origin. A critical reevaluation of the toxicity–health risk–waste management triad is needed to strengthen preventive and protective strategies in both clinical and waste-handling settings, and the review is therefore structured around targeted questions along this axis. Priority should be given to waste prevention, minimization, and segregation at source, as downstream treatment processes may introduce additional hazards. Each category of hazardous HCW exhibits specific mechanisms of toxicity, underlining the importance of targeted and informed management approaches. Future directions should include enhanced training for waste handlers, the development of unified regulatory frameworks, and improved international data collection and reporting systems. Strengthening these components is essential for reducing occupational and environmental health risks and ensuring safer conditions across healthcare systems.

## 1. Introduction

Residual materials generated from medical diagnostics, treatments, and research activities are collectively termed healthcare waste (HCW) [1]. The United Nations Basel Convention, classifies HCW as highly hazardous, ranking just below nuclear waste [2].

Diverse institutions, including hospitals, laboratories, pharmacies, research centers, dental and veterinary clinics, as well as nursing homes, generate various types of waste. Estimates suggest that approximately 15% of the HCW produced in these settings is deemed hazardous due to contamination by biological, chemical, or radioactive substances [3].

In high-income nations, hospitals typically generate up to 0.5 kg of hazardous medical waste per bed per day (kg/bed/day). In contrast, facilities in low-income countries produce approximately 0.2 kg kg/bed/day [4].

The European Union’s regulatory framework classifies HCW into three categories: infectious waste, which includes materials contaminated with pathogens; chemical waste, encompassing items containing hazardous chemicals; and pharmaceutical waste, which pertains to expired, unused, or contaminated medications and their packaging [5].

HCW generation varies significantly across continents. In Europe, Ireland leads with 7.7 kg/bed/day [5], followed by Spain at 4.4 kg/bed/day [6], while the lowest amounts are recorded in Latvia (1.18 kg/bed/day) [7] and the Netherlands (1.7 kg/bed/day) [8]. The Americas see the highest waste levels, with the United States (8.4 kg/bed/day) [6] and Canada (8.2 kg/bed/day) at the top [9], whereas El Salvador (1.85 kg/bed/day) [10] and Ecuador (2.09 kg/bed/day) [11] generate the least. In Asia, Lebanon (5.7 kg/bed/day) [12] and Kazakhstan (5.34 kg/bed/day) [7] produce the most waste, contrasting sharply with Nepal (0.5 kg/bed/day) [13] and Laos (0.51 kg/bed/day) [14], which have the lowest rates. Africa reports significantly lower HCW levels overall, with Egypt (1.03 kg/bed/day) [15] and Ethiopia (1.1 kg/bed/day) [16] at the higher end, while Mauritius (0.44 kg/bed/day) [17] and Morocco (0.53 kg/bed/day) [18] generate the least. These discrepancies highlight global differences in healthcare infrastructure, waste management efficiency, medical resource consumption, and even in the reported data.

The generation and characteristics of HCW are determined by a variety of elements, such as the type and scale of healthcare establishments, the medical interventions carried out, regional factors, economic conditions, as well as the prevailing waste management policies and procedures [11]. Other influences include the control of infections and the occurrence of epidemics. For example, an analysis conducted in Italy revealed that a significant proportion, about 52%, of all infectious medical waste comes from patients in rehabilitation services, with laboratories contributing 23%, surgeries 14%, dialysis units 7%, and emergency care 4% [6].

The risky features associated with HCW stem from one or more of the following attributes: the presence of infectious agents, genotoxic, cytotoxic, or otherwise hazardous chemicals or biologically aggressive pharmaceuticals, radioactivity, and the incorporation of used sharps [19].

Exposure to hazardous HCW poses significant risks to a wide range of individuals. This includes healthcare personnel such as doctors, nurses, and maintenance staff within medical facilities, as well as patients receiving care. Additionally, those involved in the handling or accidental exposure to such waste due to improper disposal practices are also at risk. Both healthcare workers and the general public may encounter dangers associated with HCW, emphasizing the need for stringent safety protocols and waste management practices [20].

Injuries, such as radiation burns, sharps-related wounds, and poisoning from toxic elements, effluent, or pharmaceutical residues, may occur as a consequence of exposure to HCW. For instance, the use of contaminated syringes in hazardous injection practices has resulted in the transmission of millions of new Hepatitis B virus (HBV) infections. Furthermore, waste handlers may be exposed to health risks as a result of the improper management of HCW during transportation to disposal facilities. Public health concerns are further exacerbated by the reuse of expired medications that have been improperly disposed of. Long-term health consequences, such as immune system suppression, endocrine disruption, and reproductive issues, may result from chronic exposure to specific contaminants present in HCW. Skin lesions and liver dysfunction may result from short-term, high-level exposure [2].

Ensuring the proper treatment of HCW is essential to eliminate pathogenic organisms and prevent severe health issues associated with improper waste management. Implementing effective decontamination methods safeguards public health and the environment by reducing the risks posed by hazardous waste [1].

The aim of the present narrative review is to emphasize the critical importance of awareness and management of the toxicity exerted by HCW on human health and the environment through a distinct evaluation approach focused on the HCW toxicity-health risks-waste management axis. The analysis is guided by three research questions formulated a priori. This review is guided by three main questions. First, which categories, sources, and generation patterns of HCW determine its potential to create hazardous exposures (RQ1)? Second, through which pathways and toxic mechanisms do the principal waste streams produce adverse health effects (RQ2)? And third, how can management practices be aligned with HCW within the prevention–minimization–segregation–treatment–disposal hierarchy to reduce health risks (RQ3)? Given the limited number of studies in the literature addressing HCW toxicity in detail, updating the current state of knowledge is essential. A comprehensive understanding of the hazardous effects of different HCW is crucial for improving waste-handling strategies, minimizing health risks, and developing more effective strategies for HCW management and prevention.

## 2. Methodology of Research

The present paper was methodologically designed as a narrative review targeting scientific evidence on HCW toxicity, exposure pathways, and management strategies, encompassing heterogeneous primary and secondary bibliographic sources (i.e., scientific peer-reviewed literature through reviews, original research articles and books, regulatory documents, and institutional guidelines). A narrative approach was adopted to capture the toxicological diversity of HCW streams, their health impacts, and the variability of management options, with findings integrated throughout the manuscript through a structured qualitative synthesis. The three research questions were defined a priori and guided searching, screening, and data extraction. Evidence from scientific studies were narratively synthesized and mapped to the toxicity–risk–management axis. RQ1 is addressed in Section 3, RQ2 in Section 4, and RQ3 in Section 5.

The bibliographic search was conducted across PubMed, Scopus, Web of Science Core Collection, and ScienceDirect, with Google Scholar used as a complementary source of information. Given the regulatory and operational nature of HCW, institutional and authoritative sources were also screened (i.e., World Health Organization, International Committee of the Red Cross, United Nations Environment Programme, National Institute for Occupational Safety and Health). Data on chemical–gene and chemical–disease interactions were also retrieved from the Comparative Toxicogenomics Database, an open-access, curated resource available online.

Searches covered 2000–2025, to include recent post-COVID-19 developments (e.g., circular economy approaches, emergency waste surges). Seminal pre-2000 materials were considered selectively when foundational to definitions or regulatory context. Free-text and controlled vocabulary (i.e., MeSH) with Boolean operators were combined, adapting strings per database. Core concepts and exemplars derived from the included corpus were used to ensure sensitivity across HCW categories and toxic mechanisms: (“healthcare waste” OR “medical waste”) AND (toxicity OR “human exposure” OR “health risk” OR “infectious risk” OR “endocrine disruption” OR mutagen* OR carcinogen* OR teratogen*); (“pharmaceutical waste” OR cytotoxic OR genotoxic) AND (management OR disposal OR “occupational exposure”); (mercury OR silver OR lead OR cadmium OR chlorine OR pesticide* OR “endocrine disrupting chemicals”) AND (hospital* OR clinic*); (“radioactive waste” AND medical) OR (“nuclear medicine” AND waste); (“waste management” AND (prevention OR minimization OR segregation OR incineration OR autoclave OR microwave OR pyrolysis OR gasification OR “wastewater treatment”)); (“COVID-19” AND (“medical waste” OR “PPE” OR “surge” OR “life-cycle assessment” OR “circular economy”)); (“healthcare waste” OR “medical waste” OR “hospital waste” OR “HCW generation” OR “waste generation rates” OR “waste characterization” OR “HCW types” OR “waste categories” OR “hazardous healthcare waste” OR “infectious waste” OR “pharmaceutical waste” OR “radioactive waste” OR “chemical waste” OR “HCW health risks” OR “human exposure” OR “occupational exposure” OR “environmental health risks” OR “toxic effects” OR “HCW management” OR “waste treatment” OR “waste disposal” OR “waste segregation” OR “waste minimization” OR “circular economy” OR “recycling” OR “incineration” OR “autoclaving” OR “microwave treatment” OR “pyrolysis” OR “gasification”). Given the narrative nature of this review, no quantitative pooling of study results was performed; the synthesis relies on convergent lines of evidence and biological plausibility rather than on meta-analytic effect estimates.

English-language peer-reviewed studies, book chapters from recognized publishers, and authoritative reports and guidelines addressing HCW sources, exposure routes/toxic mechanisms, and management options were included. Duplicates, non-HCW contexts, data without methodological basis and non-informative sources have been removed. Figures and tables were primarily designed for this paper, with priority given to clarity, representativeness, methodological robustness, and scientific relevance.

## 3. Categories, Origins, and Production of HCW

Addressing RQ1, Section 3.1, Section 3.2, Section 3.3 and Section 3.4 synthesize HCW categories, sources, generation rates, and composition to delineate the baseline risk context.

### 3.1. Types and Description of HCW

Around 85% of the waste generated by healthcare facilities consists of general waste, similar to household waste, and it is classified into recyclable waste, biodegradable waste, and non-recyclable waste [21]. This type of waste primarily results from administrative, housekeeping, and facility maintenance activities within healthcare settings. Typically, over half of the non-hazardous general waste consists of materials such as plastics (i.e., polyethylene terephthalate), polystyrene packaging, cardboard, and paper [22,23]. However, it does not present any distinct biological, chemical, radioactive, or physical risks [24]. The remaining 15% of waste generated by healthcare facilities is classified as hazardous, posing risks related to infection, toxicity, and, in certain cases, radioactivity [24,25].

**Figure 1:**
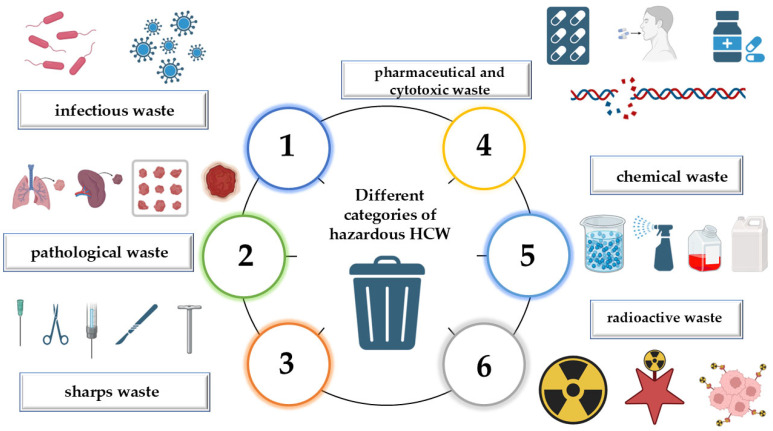
Types of hazardous HCW.

According to the World Health Organization (WHO), HCW that poses a hazard can be categorized into several types: pathological waste, infectious waste, radioactive waste, pharmaceutical and cytotoxic waste, chemical waste, and sharps waste (Figure 1) [26,27,28]. In the European Union, these WHO hazard-based types map operationally onto the European List of Waste Chapter 18 codes ((e.g., infectious ≈ 18 01 03, sharps ≈ 18 01 01 (non-infectious) or 18 01 03 (if contaminated), pathological ≈ 18 01 02, chemical ≈ 18 01 06 *, and pharmaceutical/cytotoxic ≈ 18 01 08 *) [29], while radioactive medical waste is managed under separate Euratom/national radiation rules [30]. Thus, the WHO typology and European Union coding are complementary. Each category has distinct characteristics and consequences, as outlined in Table 1.

In terms of volume, infectious waste is the predominant category of HCW, followed in smaller proportions by sharps waste (1%), body part waste (1%), chemical or pharmaceutical waste (3%), and radioactive or cytotoxic waste, including broken thermometers (less than 1%). The WHO classifies HCW based on the associated hazards. Biological (infectious) risks are linked to infectious waste, pathological waste, and sharps waste, all of which may contain pathogens capable of spreading diseases. Chemical risks are associated with pharmaceutical, chemical, and radioactive waste, which may present toxic, corrosive, reactive, or radiological dangers [21,32].

Annually, approximately 16 billion injections are administered worldwide, leading to a significant risk of improper waste management [37,38]. Not all needles and syringes are properly disposed of, which increases the potential for injury, infection, and the possibility of reuse. While the incidence of injections with contaminated needles and syringes has significantly decreased in low- and middle-income countries in recent years—due in part to initiatives aimed at curbing the reuse of injection equipment—unsafe injection practices still posed a considerable threat. In 2010, such practices were linked to around 315,000 hepatitis C infections, 1.7 million cases of hepatitis B, and approximately 33,800 new HIV infections [32,39].

Infectious waste refers to materials that are thought to contain pathogens, such as bacteria, viruses, parasites, or fungi, in concentrations or quantities sufficient to cause disease in susceptible individuals [40]. This type of waste includes items contaminated with blood or bodily fluids, as well as cultures and stocks of infectious agents from laboratory activities. It also includes waste generated by patients isolated due to infections. Laboratory cultures and stocks are considered particularly hazardous as they are highly infectious. Additionally, waste from autopsies, animal carcasses, and any materials that have been infected, inoculated, or come into contact with highly infectious agents are classified as highly infectious waste [24].

Pathological waste is sometimes evaluated as a subset of infectious waste; however, it is frequently categorized independently, particularly when specific procedures for its management, treatment, and disposal are required [41].

Pharmaceutical waste encompasses medications that are expired, unused, spilled, or contaminated, as well as prescribed and over-the-counter drugs, vaccines, and serums that are no longer needed [42]. Due to their chemical or biological properties, these substances must be disposed of with great caution [24]. Improperly disposed unused pharmaceutical chemicals and their degradation products contribute to environmental contamination, adversely affecting human health and both terrestrial and aquatic ecosystems [43,44]. Furthermore, pharmaceuticals have been found in various environmental compartments, including groundwater, surface water, and soil. Environmental samples typically contain a variety of pharmaceuticals, including antibiotics, antidepressants, lipid-lowering agents, beta-blockers, and hormones. These substances can accumulate in ecosystems due to human activities, posing potential risks to both environmental and human health [45,46].

Cytotoxic waste originates from various sources and may include the following: materials contaminated during the preparation and administration of drugs, such as syringes, needles, gauzes, vials, and packaging; expired medications, surplus (leftover) solutions, and drugs returned from patient care areas; as well as urine, feces, and vomit from patients that could contain harmful quantities of administered cytostatic drugs or their metabolites. These substances should be regarded as genotoxic for a minimum of 48 h, and in some cases, up to one week after the administration of the medication [47]. Widely recognized genotoxic substances, which have been classified as carcinogenic by the working groups of the International Agency for Research on Cancer, include azathioprine, ciclosporin, cyclophosphamide, and tamoxifen, etc. [24,48].

**Figure 2:**
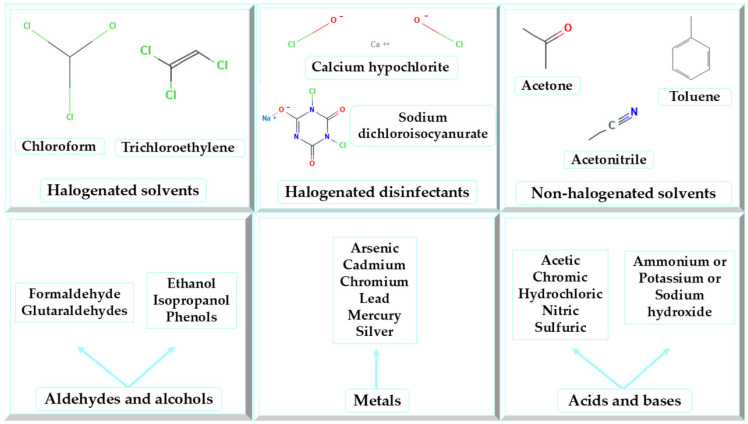
Chemical wastes from healthcare activities.

Numerous chemicals and pharmaceuticals utilized in healthcare settings pose potential hazards [49]. Chemical waste can have detrimental effects on human health, often leading to poisoning as the primary outcome upon exposure. Healthcare-related chemical waste is classified as hazardous if it exhibits at least one of the following characteristics: toxicity, corrosivity (e.g., acids with pH ≤ 2 or bases with pH ≥ 12), flammability, reactivity (including explosiveness, sensitivity to shock, or reactivity with water), or oxidizing properties [1]. Figure 2 illustrates the chemical waste generated by healthcare operations [24,50].

Radioactive waste consists of materials contaminated with radionuclides or radioisotopes. These wastes are produced through various investigative and therapeutic practices, in vivo organ imaging and tumor localization, and in vitro analysis of body tissue and fluid [51].

### 3.2. Sources of HCW

HCW originates from a wide range of medical facilities and activities, including hospitals, clinics, diagnostic laboratories, and research institutions. Additionally, it is produced in blood banks, pharmacies, nursing homes, veterinary testing centers, autopsy units, home-based treatments, ambulance operations, funeral services, etc. HCW may vary by source, as outlined in Table 2 [24,52,53,54].

In the absence of explicit regulations and standardized directives, these categorizations may vary slightly depending on the reference source. The WHO primarily distinguishes between major and minor sources [24], whereas another comprehensive source classifies them into large, medium, and small categories [54]. Proper waste classification should rely on a thorough understanding of disease propagation mechanisms and risks associated with hazardous chemical exposure.

Small-scale and dispersed sources generate HCW, though the volume and composition can differ. These sources share certain characteristics: sharp objects are primarily limited to hypodermic syringes, human anatomical waste is typically absent, and they infrequently produce cytostatic or radioactive materials [55].

### 3.3. Generation of HCW

The generation of HCW varies significantly across countries and is influenced by several elements. Key factors contributing to this variability include the waste management practices implemented, the nature and specialization of healthcare establishments, the availability of reusable medical equipment, and the daily patient volume handled by these facilities Metropolitan general hospitals (10.7 kg/bed/day) and laboratories (7.7 kg/bed/day) produce the highest total HCW, while the highest infectious waste generators are metropolitan general hospitals (2.79 kg/bed/day) and rural general hospitals (2.03 kg/bed/day). The lowest total HCW producers are veterinarian (individual, metropolitan) (0.65 kg/bed/day) and doctor’s office (rural) (0.93 kg/bed/day). The smallest infectious waste generators are psychiatric hospitals (0.043 kg/bed/day) and nursing homes (0.038 kg/bed/day) [56].

In comparison to developed nations, HCW generation tends to be lower in developing countries. The most reliable data on HCW production is acquired through comprehensive quantitative waste assessments. Such assessments involve setting clear objectives, detailed planning, engaging staff members, acquiring necessary equipment (e.g., scales, protective gear), gathering and analyzing data, and offering actionable recommendations. This evaluation process not only allows for the enhancement of existing waste management practices but also raises awareness among healthcare professionals about waste-related issues and identifies opportunities for waste reduction. Additionally, effective waste segregation can prevent the overuse of resources, leading to cost savings [57,58,59].

Table 3 presents data on HCW production, including the type of facilities and the corresponding quantities of infectious waste generated across various countries, ranging from the highest to the lowest producers. However, the correlation between the generation of HCW and patient safety remains unclear [57].

In Pakistan, maternities are the largest generators of HCW overall, producing 4.1 kg/bed/day of total HCW, and 2.9 kg/bed/day of infectious waste. For hospitals, the total HCW generation is 2.07 kg/bed/day, with a range of 1.28–3.47 kg/bed/day, but there is no specific data available for infectious waste generation in this category. The smallest producers of total HCW are consulting clinics, with 0.025 kg/bed/day, followed by basic health units, which generate 0.04 kg/bed/day. For infectious waste, consulting clinics contribute the least with 0.002 kg/bed/day, and basic health units generate 0.03 kg/bed/day. This shows a clear difference in waste generation between large facilities such as maternity homes and smaller healthcare institutions like consulting clinics and basic health units [54,60,61].

For Tanzania, the total HCW generation across the various healthcare establishments sums up to 0.21 kg/bed/day, while the total infectious waste generation equals 0.117 kg kg/bed/day.

Among the different sources, hospitals are the largest contributor to total HCW with 0.14 kg/bed/day, followed by rural dispensaries with 0.04 kg/bed/day patient per day. On the other hand, health centers in urban areas generate the least amount of both total and infectious waste, producing 0.01 kg/bed/day for total HCW and 0.007 kg/bed/day for infectious waste [54,62].

In South Africa, the Provincial tertiary hospital generates the most infectious waste, with 1.53 kg/bed/day, while the Public clinic produces the least, with only 0.008 kg/bed/day [54,63].

Reports indicated that overall HCW production is lower in developing nations. However, this reduction in waste generation does not inherently reflect a higher level of patient safety within healthcare facilities.

The global mass of HCW is consistently rising by 2–3% annually, a trend further intensified by the COVID-19 pandemic [74,75,76]. The data showed significant differences in HCW generation during COVID-19, reflecting short-term surge conditions rather than baseline operations [77,78,79,80], with Japan producing the highest amount at 876 tonnes/day, followed by India with 608 tonnes/day and Indonesia with 290 tonnes/day. In contrast, Afghanistan, Mexico, and Nepal recorded much lower levels, ranging between 27 and 37 tonnes/day. During the COVID-19 pandemic, HCW generation surged dramatically across major cities [81]. In Manila, with a population of 14 million, daily waste production rose from 47 to 280 tonnes/day, reflecting an increase of 496%. Similarly, Jakarta experienced a spike from 35 to 212 tonnes/day, marking a 506% rise. Bangkok also saw a sharp increase, with waste jumping from 35 to 210 tonnes/day (500%). Even in smaller cities like Ha Noi and Kuala Lumpur, where pre-pandemic levels were around 26–27 tonnes/day, waste generation grew by over 490%, highlighting the immense strain on waste management systems, underscoring that these pandemic spikes are outliers and should not be generalized to non-pandemic years [82].

### 3.4. HCW Composition

Analyzing the composition of HCW helps identify the most common waste types, which in turn informs the development of more effective treatment and recycling methods. Different materials, such as plastics, paper, metals, and biological waste, require distinct disposal and treatment methods [83].

In Jordan, the primary component of HCW is paper (38%), followed by plastics (27%), glass (10%), metals (5%), textiles (11%), and organics/food waste (17%). The significant presence of recyclables suggests opportunities for improved waste segregation and material recovery. This indicates that better waste management strategies could lead to more efficient recycling practices and reduce landfill dependency [84,85].

In Peru, HCW composition includes paper (22% mixed, 5% cardboard), plastics (12%), glass (8%), metals (2%), textiles (–), and organics/food waste (15%). Anatomical waste is minimal at 0.1%, while “other” waste accounts for 27%, indicating potential challenges in categorization and separation and highlighting the need for clearer waste classification systems [24,86].

In Turkey, HCW is dominated by plastics (41%), followed by paper (16%), glass (7%), metals (4%), textiles (10%), and organics/food waste (12%). The high plastic content underscores the need for waste reduction efforts and sustainable disposal strategies, with an emphasis on reducing plastic use and promoting alternatives to enhance environmental sustainability [24,87].

In Taiwan, the composition shows paper and cardboard (34%), plastics (26%), glass (7%), metals (9%), textiles (9%), and organics (–). The balance of materials suggests a mix of recyclable and non-recyclable waste, requiring efficient waste handling and recycling systems to minimize environmental impact and increase material recovery rates [24,25].

In Kuwait, HCW includes paper (24%), plastics (18%), glass (10%), metals (0.4%), textiles (–), and organics/anatomical waste (8%), with an additional 8% classified as “other.” The anatomical fraction requires strict biohazard management protocols [24,88,89].

In Italy, HCW consists mostly of plastics (46%), followed by paper (34%), glass (8%), metals (–), textiles (11%), and liquids/organics (12%). Textiles and liquids make up 11% and 12%, respectively, with a minimal metal presence. The predominance of plastics and paper suggests a need for targeted recycling programs to reduce environmental impact and promote sustainability through material recovery and alternative solutions [90].

Comprehending the varied composition of HCW across nations underscores the necessity for customized waste management techniques that emphasize recycling, waste minimization, and specialized disposal to alleviate environmental hazards, foster sustainability, and prevent health risks.

## 4. Health Hazards of Toxic HCW Exposure

HCWs are definitive sources and causes of toxicity within the human body, indicating significant health risks in various contexts. These risks arise from improper waste management, inadequate handling, and unsafe disposal practices, posing serious threats to biomedical staff, patients, and sanitation workers involved in disposal. Exposure can lead to environmental contamination, occupational hazards, and long-term public health consequences [2]. Addressing RQ2, Section 4 maps exposure pathways and toxic mechanisms by stream, emphasizing sharps/infectious, pharmaceutical/cytotoxic, chemical, and radioactive wastes.

### 4.1. Individuals Potentially Exposed to HCW Toxicity

Anyone who comes into contact with hazardous HCW faces potential risks of toxicity, including those involved in its generation, handling, or accidental exposure due to negligent practices [91].

The primary at-risk groups include medical professionals such as doctors, nurses, and auxiliary staff, as well as hospital maintenance workers. Patients in medical facilities, individuals receiving home-based care, and visitors are also vulnerable. Additionally, personnel responsible for waste-related support services—such as cleaners, laundry staff, and porters, along with those transporting, treating, or disposing of waste, including landfill workers and informal waste pickers, face significant health hazards. The broader population may also be endangered when hazardous medical waste is abandoned or disposed of improperly. Special attention must be given to dispersed sources, including pharmaceutical and infectious waste from home healthcare, such as dialysis disposables, used insulin needles, and contaminated materials from illicit intravenous drug use [92,93,94].

Effective medical waste management within healthcare facilities relies on a well-structured waste management team, efficient administration, and meticulous strategic planning. The implementation of legal regulations, sufficient financial resources, and the active engagement of properly trained staff are also essential to ensuring a safe and sustainable waste disposal process [95]. It is essential that comprehensive education and training programs are accessible to all individuals involved in the segregation and collection of waste, ensuring that they are well-equipped with the necessary knowledge and skills to handle these processes efficiently and in accordance with established guidelines [96,97].

### 4.2. Toxicity Risks to Health

Numerous nations in the process of development, as well as underdeveloped urban areas, do not have established protocols or frameworks for the secure handling of HCW. The improper management and disposal of medical waste are prevalent, resulting in significant health hazards, which are closely linked to the specific nature of the waste involved [98].

Hazardous HCW can pose serious threats, including bacterial contamination, radiation exposure leading to skin and respiratory damage, toxic poisoning, and environmental pollution. Improper disposal of waste in landfills can also jeopardize the water supply and disrupt ecosystems. HCW is associated with several known health risks, which can affect the general population in several main ways: exposure to toxic substances from the waste incineration processes, contamination of water sources with harmful chemicals or pathogens, and accidental contact with waste at public disposal sites [54].

#### 4.2.1. Toxicity from Sharps, Pathological and Infectious Waste

Infectious waste should always be regarded as a potential carrier of pathogenic microorganisms, as it is impossible to determine at the time of disposal whether a particular item contains harmful agents. If not properly managed, infectious waste can facilitate pathogen transmission through multiple exposure pathways. These include direct entry into the body via skin injuries such as cuts, abrasions, or punctures, absorption through mucous membranes, inhalation of airborne contaminants or accidental ingestion [99].

HCW has the potential to spread over 30 hazardous bloodborne pathogens, posing a significant risk of infection if not properly managed [100]. The spread of infections and the strategies for their prevention can be understood through the concept of the “chain of infection,” which consists of several interconnected components: the infectious agent, a reservoir (such as humans), an exit pathway, the mode of transmission, an entry point, and a vulnerable host. Among the various types of hazardous HCW, highly concentrated pathogen cultures and contaminated sharps—especially hypodermic needles—represent some of the most critical threats to health, as they significantly increase the risk of direct exposure to infectious agents [101].

**Figure 3:**
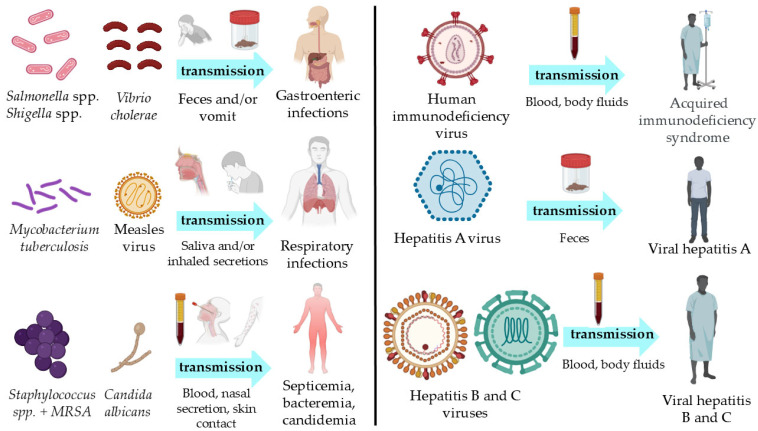
Examples of pathogenic bacteria (*Salmonella* spp., *Shigella* spp., *Vibrio cholerae*, *Mycobacterium tuberculosis*, *Staphylococcus* spp. including methicillin-resistant *Staphylococcus aureus*), viruses (measles virus, human immunodeficiency virus, hepatitis A, B, and C viruses), and fungi (*Candida albicans*) potentially linked to healthcare waste. The figure illustrates their main transmission routes (i.e., feces, vomit, saliva, inhaled secretions, blood, body fluids, nasal secretions, skin contact) and the associated infections (gastroenteric, respiratory, septicemia, bacteremia, candidemia, acquired immunodeficiency syndrome, and viral hepatitis). MRSA, Methicillin-resistant *Staphylococcus aureus*.

Figure 3 presents notable examples of common and significant viral, bacterial, and fungal infections that can arise from exposure to HCW.

A major concern regarding HCW is the risk of transmission of bloodborne viruses, particularly HIV, HBV and Hepatitis C virus (HCV). Strong evidence indicates that these infections can spread through injuries caused by syringe needles contaminated with infected blood, especially when sharps waste is not properly managed. While any needlestick injury has the potential to transmit bloodborne pathogens, research suggests that hollow-bore needles present a higher risk of infection compared to solid needles [102,103].

Sharps pose a dual hazard, as they can not only cause mechanical injuries but also serve as a vehicle for infection if contaminated with harmful microorganisms. The primary concern is the potential for pathogen transmission through percutaneous exposure, where infectious agents enter the body via puncture wounds, increasing the risk of disease [104].

An alarming estimation showed that 600,000 to 800,000 needlestick and other percutaneous injuries occurred annually in the United States. In the United Kingdom, events averaged 100,000 each year. Up to 30% of Hepatitis B, 1–3% of Hepatitis C, and 0.3% of HIV infections have been connected to improper HCW disposal [105].

A medical investigation indicated that the prevalence of HBV and HCV was markedly elevated among persons managing medical garbage in contrast to those handling non-clinical waste [106]. Moreover, it is estimated that up to 5% of HIV infections in Africa arise from hazardous injecting practices, such as being exposed to sharps injuries due to inappropriate management of medical waste [107].

Such injuries additionally increase the probability of negative health outcomes and also induce anxiety, fear, and emotional distress among medical personnel [108]. Thus, it may be inferred that HCW poses a considerable health hazard to individuals employed in medical settings [109,110].

Among the most critical waste categories generated by major sources of HCW generation (i.e., hospitals, medical centers, laboratories, vaccination campaigns, radiology units) are sharps, which include hypodermic needles, intravenous sets, scalpels, blades, broken vials and ampoules, as well as broken glass and pipettes. These materials pose a significant risk of injury and infection due to their ability to puncture the skin and potentially transmit pathogens. Additionally, infectious and pathological waste is a substantial concern in healthcare settings, encompassing items such as dressings, bandages, gauze, and cotton contaminated with blood or bodily fluids, as well as gloves, masks, suction canisters, and surgical gowns exposed to infectious agents. This category extends to biological waste, including blood and other bodily fluids, microbiological cultures, infected animal carcasses, tissues, organs, and even body parts, all of which require specialized handling and disposal to prevent contamination and disease transmission [24].

Beyond major healthcare institutions, minor sources such as physicians’ offices, dental clinics, and home healthcare services also contribute to HCW, albeit on a smaller scale. These facilities generate sharps waste, which primarily consists of needles, syringes, lancets, broken ampoules, and insulin injection needles—items that can still pose a risk of needlestick injuries and pathogen transmission. Additionally, infectious and pathological waste from these minor sources includes materials such as cotton, gauze, dressings, gloves, and masks contaminated with blood or bodily fluids [24,31].

#### 4.2.2. Toxicity from Pharmaceutical and Genotoxic Waste

Pharmaceuticals utilized in healthcare are often categorized as hazardous substances. While they typically exist in minimal amounts within HCW, larger quantities are usually generated when expired or unneeded medications are disposed of [43,111,112]. Among the most common types of waste are pharmaceutical and genotoxic/cytotoxic waste, which include expired drugs, spilt drugs, bulk chemotherapeutic waste, vials, gloves, and other materials contaminated with cytotoxic agents, as well as contaminated excreta and urine [24]. The management of pharmaceutical waste presents a complex challenge due to the substantial quantities stored in pharmacies, hospitals, distribution centers, and other facilities. Effective control measures are essential to prevent leakage or accidental public exposure to these substances [54].

The prolonged presence of pharmaceuticals in the environment can lead to both immediate and long-term effects, including acute toxicity, chronic health issues, alterations in behavior, reproductive dysfunctions, and suppression of cellular growth in both humans and animals [113,114].

Pharmaceuticals infiltrate ecosystems predominantly through the incorrect disposal of unwanted or expired prescriptions, frequently discarded via sewage systems. These compounds have been detected in multiple environmental compartments, such as groundwater, surface waterways, and soil. Frequently identified types of pharmaceuticals in these environmental samples encompass beta-blockers, antidepressants, hormones, lipid-lowering medicines, antibiotics, and non-steroidal anti-inflammatory drugs [45,46].

The excessive and inappropriate use of antibiotics for bacterial infections, coupled with the improper disposal of pharmaceutical waste containing non-metabolized antibiotics and their byproducts, is leading to elevated levels of antibiotics in the environment, resulting in ‘antibiotic pollution’. This type of pollution is emerging as a significant global concern, as it is a principal contributor to the emergence of antibiotic and antimicrobial resistance, which poses severe implications for public health, resulting in an increase in human diseases [115].

The inappropriate disposal and misuse of antibiotics in trash lead to the emergence of antibiotic-resistant microorganisms in the environment. These resistant organisms can disrupt the balance of the human gut microbiome, allowing harmful pathogens to proliferate. This imbalance can lead to a variety of health concerns, including gastrointestinal disorders and an increased risk of diseases such as colorectal cancer. As these resistant bacteria evolve, they may become superbugs, which are difficult or impossible to treat, posing a serious threat to human health, including potentially fatal infections [116].

The growing presence of estrogens and xenoestrogens in the environment is becoming a significant issue, as their levels rapidly rise due to improper disposal of waste [117]. These estrogenic substances pose significant risks to both human health and ecosystems, as they can easily migrate across different environmental media, such as water, soil, and air [118]. Such compounds exert a wide range of detrimental effects on both human and animal health, influencing various physiological processes such as hormonal balance, nervous system functions immune, and cardiovascular. Therefore, it is crucial to implement effective and sustainable approaches to manage these compounds, especially in aquatic environments, to mitigate their impact [119].

Additionally, studies have linked exposure to external estrogens with a heightened risk of developing breast cancer among women in Spain [120]. Studies have highlighted a correlation between increased levels of urinary phytoestrogens and idiopathic infertility in men residing in China, suggesting a potential reproductive health risk [121].

Collectively, the findings from these studies indicate that the contamination of surface waters by estrogens can have detrimental effects on the reproductive health and fertility of both humans and wildlife across the globe [122].

Genotoxic waste comprises very hazardous substances, including chemotherapeutic agents and their metabolites, as well as mutagenic, teratogenic or carcinogenic compounds [40].

The principal modes of exposure to genotoxic waste are inhalation of airborne particles and dermal absorption. Nonetheless, ingestion, inadvertent needle-stick injuries, or other puncture wounds present possible hazards. Moreover, exposure may arise from contact with bodily fluids and secretions, including vomit, urine, or feces, from individuals receiving chemotherapy [47,123].

The cytotoxic effects of diverse antineoplastic medicines frequently rely on the cell cycle, specifically targeting stages like DNA replication and cell division. Conversely, some medications, such as alkylating agents, are non-phase specific and can induce toxicity during any phase of the cell cycle. Research has demonstrated that several antineoplastic agents possess carcinogenic and mutagenic characteristics, with certain chemotherapy regimens associated with the emergence of secondary malignancies, perhaps arising subsequent to the effective treatment of the primary malignancy [47,124].

**Figure 4:**
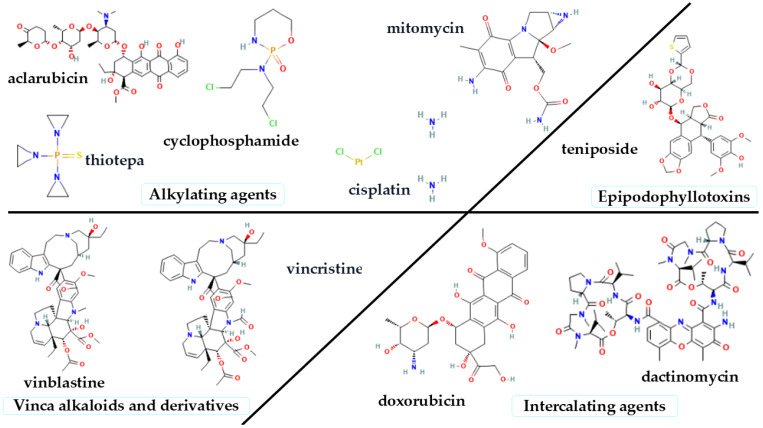
Classification and chemical structures of hazardous cytotoxic drugs.

Figure 4 illustrates the classification and chemical structures of cytotoxic drugs hazardous to the eyes and skin, including alkylating agents, intercalating agents, vinca alkaloids and derivatives, and epipodophyllotoxins, highlighting both vesicant and irritant drugs within each category [24,50].

Cytotoxic medicines exhibit potent irritating qualities, and direct contact can result in localized symptoms, including rash, dermatitis, skin irritation, mucous membrane ulcers, and irritation of the throat or eyes [125]. The adverse effects from extended or recurrent exposure to cytotoxic agents are considerable and grave. An elevated occurrence of spontaneous abortions during gestation and congenital abnormalities has been noted in offspring of women with previous records of occupational exposure to antineoplastic agents [126]. Furthermore, cytotoxic medications are not environmentally neutral, particularly concerning aquatic ecosystems; thus, any release of genotoxic waste could result in catastrophic ecological repercussions [127].

#### 4.2.3. Toxicity from Chemical Wastes

Exposure to chemical waste can have detrimental effects on human health, often leading to poisoning as the main consequence of direct contact. Both short-term (acute) and long-term (chronic) exposure can lead to intoxication, while physical harm, such as chemical burns, is frequently observed. Poisoning may occur through various routes, including skin absorption, inhalation, ingestion, or contact with mucous membranes. Chemicals that are flammable, corrosive, or reactive, such as formaldehyde and other volatile compounds, can cause injuries when they come into contact with the skin, eyes, or respiratory passages [24,128].

In research and specialized hospitals, laboratory staff are regularly exposed to various hazardous substances. These include batteries, spent disinfectants, broken thermometers, and discarded medications. Additionally, chemicals such as methylene chloride, xylene, toluene, methanol, and formalin are commonly found. Other forms of waste include lubricants, cleaning solvents, oils, asbestos, and waste anesthetic gases. Broken mercury devices, blood pressure gauges, and other dangerous materials are also part of the chemical waste regularly handled by laboratory personnel [24].

Recognizing the risks associated with specific chemicals or substances is straightforward by proper labeling. This includes symbols, warning labels, and hazard statements, all of which follow the guidelines set by European and international standards, including the Globally Harmonized System [31].

The disposal of chemical waste presents considerable dangers to both human health and the environment due to its harmful characteristics. Numerous chemicals are hazardous, and their fumes, dust, or vapors can easily enter the bloodstream through inhalation, potentially causing severe health [129].

Substances with corrosive properties, such as strong acids and alkalis, have the potential to inflict serious burns and irreversible damage to the skin and eyes. Additionally, certain chemicals may decompose into hazardous gases, further amplifying their level of danger [130,131].

Chemicals that are flammable, such as solvents and fuels, can catch fire with little provocation, quickly spreading flames and generating intense heat, especially in confined spaces. In addition, some reactive substances can ignite spontaneously when exposed to air or water, releasing hazardous gases. These dangerous traits emphasize the necessity for careful management and disposal of chemical waste to limit exposure and prevent adverse effects [132,133].

The toxicity of non-essential heavy metals differs for each element. While aluminum, lithium, tin, and barium are considered to have lower toxic effects, highly hazardous metals such as mercury, cadmium, arsenic, and lead are known to cause serious health complications [134,135].

Mercury in HCW readily volatilizes at ambient temperature and pressure, remaining in the atmosphere for approximately one year. Mercury is toxic in both its elemental and methyl forms. It can be lethal if breathed or contacted. When inhaled, 80% of mercury vapor enters the circulation through the lungs, damaging the neurological, digestive, respiratory, immunological, and kidney systems. Exposure to mercury can result in tremors, visual and auditory impairment, paralysis, insomnia, mood fluctuations, prenatal defects, and attention or cognitive deficiencies in children [136].

Mercury is commonly found in various medical instruments, particularly in devices such as blood pressure monitors and fever thermometers. The presence of this toxic metal poses significant risks, especially when these instruments break or require proper disposal [137,138]. In HCW, one of the most recognized sources of mercury is batteries, particularly the small button-type variants [139]. In developed nations, the primary contributor to mercury exposure in both the adult and fetal central nervous system is mercury vapor emitted from dental amalgam [140].

The presence of mercury in medical applications is gradually declining. In contrast, silver, a metal with potential toxicity, is being incorporated into a growing number of uses, including its role as an antimicrobial agent and in nanotechnology. Excessive exposure to silver can cause irreversible discoloration of the skin, turning it permanently gray. Regulatory bodies and experts are increasingly concerned about its possible health effects, including the risk of systemic complications and the development of argyria [111,141].

Healthcare facilities utilize significant amounts of disinfectants, including chlorine and quaternary ammonium compounds, both of which have corrosive properties. It is important to recognize that such reactive substances can produce highly toxic secondary compounds. When chlorine is applied in poorly ventilated areas, its interaction with organic matter can lead to the release of chlorine gas as a hazardous by-product. To minimize risks, strict safety protocols should be followed to prevent air concentrations from surpassing acceptable exposure limits [24,142].

Obsolete pesticides, such as aldrin, mirex, lindane, hexachlorobenzene, toxaphene, dieldrin, endrin, DDT, kepone, chlordane, and heptachlor, when contained in compromised vessels such leaking drums or shredded bags, present direct or indirect hazards to human health. During substantial precipitation, these substances may infiltrate the soil and pollute groundwater. Contact with these compounds may occur by dermal exposure to the pesticide, inhalation of fumes, or ingestion of contaminated water or food. Further dangers include the possibility of spontaneous combustion due to incorrect storage and environmental pollution resulting from inadequate disposal procedures, such as incineration or burial of the chemicals. These herbicides are recognized for inducing oxidative stress, disrupting hormone balance, and modifying gene expression, potentially resulting in severe health issues, even malignancies [143,144].

The Comparative Toxicogenomics Database is an informational tool that illustrates the correlations between chemicals, diseases, and gene networks. The evidence shows that chemicals not only pose immediate health hazards but can contribute to the development of chronic conditions, including various cancers, metabolic disorders, and neurodegenerative diseases. Table 4 presents potential associations between exposure to various chemicals and the diseases most likely to develop or worsen as a result. A high inference score indicates that the chemical may play a more significant role in the development or exacerbation of that specific disease. Chemical wastes, such as mercury, silver, chlorine, and various pesticides, are strongly correlated with a range of health risks, including serious diseases and disorders. For instance, mercury exposure is associated with conditions like type 2 diabetes, autism spectrum disorder, and obesity. Silver, often used in medical applications, has been linked to nerve degeneration, diabetes, and chemical-induced liver injury. Pesticides like aldrin and mirex are particularly concerning due to their strong association with different forms of cancer, including liver and prostate neoplasms, as well as skin cancers. Endrin exposure is also connected to adenocarcinomas and breast cancer. Additionally, chemicals like chlorine are related to respiratory issues such as pulmonary fibrosis, acute lung injury, and myocardial ischemia [145].

#### 4.2.4. Toxicity from Radioactive Wastes

Hazardous waste containing radioactive substances falls under the category of radioactive waste. This includes materials that possess inherent radioactivity as well as those that have been exposed to radioactive contamination and are no longer deemed useful [36,146]. Sectors involved in generating radioactive waste include nuclear medicine, where highly active sources are utilized, particularly in diagnostic equipment such as sealed sources containing gallium [24].

Medical facilities are increasingly relying on radioactive isotopes for both diagnostic procedures and therapeutic treatments. The most utilized radioisotopes in healthcare settings include various iodine isotopes (131, 125, and 123), carbon-14, technetium-99m, and fluorine-18. Radioactive solid waste typically consists of medical items contaminated with trace amounts of radioactivity, such as syringes, needles, cotton swabs, vials, gloves, and absorbent materials. Additionally, clothing and personal items from patients who have received high doses of iodine-131 contribute to radioactive waste. Most radioactive waste produced in hospitals falls into the low-level category, with occasional medium-level waste containing isotopes with relatively short half-lives [147].

Health effects resulting from exposure to radioactive waste depend on both the intensity and duration of contact with radiation. Symptoms can vary widely, from mild conditions such as dizziness, nausea and headaches to severe complications, including extensive tissue damage and cellular destruction [24,148].

#### 4.2.5. Toxicity from HCW Management Practices

Beyond the distinct dangers linked to various categories of HCW, occupational risks also arise from handling and treating such materials. The improper disposal and inadequate processing of hazardous medical waste can further amplify these threats, endangering workers involved in waste management [31,149].

Emissions released from waste incinerators can affect individuals residing or working near treatment facilities. The health risks become particularly severe when incinerators are inadequately maintained or improperly operated. Without effective control measures, pollutants generated during the incineration process, especially particulate matter, can raise significant health concerns, contributing to an increased incidence of respiratory and cardiovascular diseases [150]. Highly volatile metals, including mercury and cadmium, have been linked to adverse effects on the neurological and immune systems, as well as causing damage to the kidneys and lungs. Additionally, compounds such as dioxins, furans, and polycyclic aromatic hydrocarbons are recognized carcinogens, but their harmful impact extends beyond cancer, potentially leading to other severe health complications [151].

Toxic and hazardous by-products such as dioxins, furans, and co-planar polychlorinated biphenyls originate from various industrial activities, often released as emissions or fly ash when incineration occurs at temperatures below 800 °C. While the toxicity of dioxins and furans varies, these compounds are highly persistent, resisting degradation and accumulating within the food chain over time [2,152,153].

Water that remains after waste treatment contains both organic and inorganic pollutants. It is essential to regularly assess contaminant levels to ensure that any discharged effluent complies with established regulatory standards before entering sewage networks [154].

The disposal of HCW in landfills and incinerators presents potential risks to both the environment and nearby populations. Landfilling such waste could expose workers and the surrounding public to hazardous conditions. Smoke emitted from waste incineration may contain toxic substances, including heavy metals, which pose long-term health risks to those working at the site and to the general community. Evidence consistently suggests that proximity to specialized waste landfills is associated with an increased likelihood of congenital disorders and a higher rate of hospitalizations due to respiratory illnesses [155,156].

#### 4.2.6. Mechanism of Toxic Action

Human contact with HCW has been linked to elevated concentrations of various hazardous chemicals. The growing body of research highlighting distinct and significant health risks associated with HCW exposure has fueled an increase in risk assessment studies and awareness initiatives. Although extensive scientific data exist regarding the adverse health effects of many well-documented HCW-related chemicals, there remains a considerable gap in understanding their impact at the molecular and cellular levels. Furthermore, the toxicological consequences of complex chemical mixtures encountered during and after informal HCW recycling, along with their precise mechanisms of action, are still largely unexplored [129,157].

Table 5 outlines the toxic mechanisms of action for the most common HCW substances that pose health risks, as discussed in the subsections of Section 3.

## 5. HCW Management

In response to RQ3, Section 5 connects stream-specific hazards with appropriate prevention, minimization, and treatment options, while also summarizing the current knowledge base on waste management technologies.

Effective management of HCW requires organization and strategic planning at the local, regional, and national scales. Safeguarding public health through proper waste handling can be accomplished using various approaches. These methods are generally ranked in a hierarchy, often referred to as the “waste hierarchy,” which prioritizes actions starting with waste prevention, followed by reduction, reuse, recycling, recovery, treatment, and finally disposal [24].

The waste management hierarchy is primarily founded on the principles of the “3Rs” (i.e., reduce, reuse, and recycle), which are essential for promoting the efficient and sustainable use of resources. This framework encourages practices that minimize waste generation, prioritize reusing materials, and enhance recycling efforts, all of which contribute to resource conservation and environmental sustainability [171].

Minimizing and reducing health risks associated with HCW starts with the prevention of waste generation. The most successful strategy for waste management is fundamentally to prevent waste generation by abolishing wasteful behaviors. Significant waste reduction necessitates coordination with medical professionals to alter clinical practices, hence minimizing material usage. Practices that facilitate waste minimization encompass: selecting products that generate less waste, such as those utilizing smaller quantities or yielding fewer hazardous byproducts; employing physical cleaning methods like steam sterilization instead of chemical agents; curbing the overuse of supplies, particularly in nursing and sanitation activities; procuring smaller quantities more frequently rather than making large bulk purchases, especially for perishable goods; prioritizing the use of older stock; and maximizing the utilization of every container’s contents prior to disposal [24,172,173].

Reprocessing and reusing healthcare products and supplies are prevalent waste management strategies aimed at enhancing economic and environmental advantages [174]. As long as proper cleaning reduces infection risks, reusable medical equipment should be favored. It is important to distinguish between non-medical disposable goods, medical instruments that do not cross-contaminate, and devices designed for repeated use, like surgical tools, when assessing the feasibility of reusing items. Furthermore, Certain categories of non-disposable medical devices, such as endoscopes, are reused by approximately 41% of Canadian hospitals. Devices that are designated as “reusable” are eligible for reuse [24]. Nevertheless, the risk of disease transmission is considerable, and syringes, hypodermic needles, and catheters should not be reused [175]. Reuse may necessitate any or all of the subsequent procedures: decontamination, disinfection, cleansing, sterilization, and reconditioning [176].

Recycling has become more prevalent in healthcare environments, particularly due to the significant volume of non-hazardous refuse [44]. This method has the potential to result in substantial cost reductions, either by reducing disposal expenses or by receiving payments from recycling companies for recovered materials. In comparison to alternatives such as steel, ceramics, and glass, plastic is the preferred material for numerous medical instruments due to its affordability, durability, and flexibility. Consequently, it is imperative to recycle plastic-based materials. Furthermore, other non-plastic waste materials have been identified as recyclable, such as stainless steel from surgical instruments, medical implants, and aluminum from discarded pharmaceutical blister packs [174].

Energy recovery is the most common definition of “recovery”, which involves the conversion of refuse into fuel for the purpose of generating electricity or direct heating [24].

Quaternary recycling, or energy recovery, uses waste incineration energy but emits toxic gases, making it unsuitable for medical waste recycling. HCW incinerators are too tiny to recover energy efficiently. Reusing incineration byproducts reduces its environmental impact. Swapping fine aggregates in concrete with biomedical waste ash increases its strength and decreases its permeability while reducing the need to dump the ash in landfills [174].

HCW treatment options are diverse, but conventional methods face limitations such as extended processing times, byproduct generation, high energy demands, and high costs, necessitating the development of more efficient technologies. Steam sterilization effectively deactivates pathogens at low temperatures but is unsuitable for pharmaceutical, radioactive, and pathological waste. Microwave radiation kills microorganisms at 200 °C, and reverse polymerization in nitrogen-rich environments decomposes organic compounds without combustion. Hot air ovens, utilizing conduction and convection, provide more effective disinfection than steam. Carbonization of polymers produces activated carbon and nanotubes. Converter technologies reduce waste volume by combining pasteurization, sterilization, and grinding. Bio converters use enzymes to sterilize waste, producing sludge for disposal. Incineration reduces waste by up to 90%. Oxygen-deprived pyrolysis converts waste into char, oil, and syngas, while gasification in oxygen-limited conditions generates syngas. Irradiation with UV or gamma radiation disrupts microorganism DNA. Chemical disinfection destroys pathogens, while radioactive waste is encapsulated in cement or plastic to prevent dispersion. Landfill monitoring is critical due to environmental risks. Procession utilizes freezing and vibration to decompose waste into fine particles, and thermal plasma technology transforms waste into harmless substances by heating inert gases. Advances in thermal technologies could replace incineration with waste-to-energy solutions [177].

**Figure 5:**
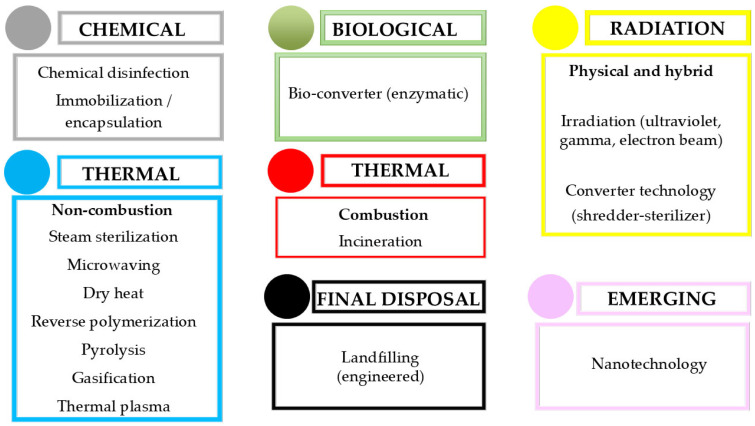
Comparative description of various HCW treatment methods, highlighting their respective advantages and disadvantages.

Figure 5 illustrates the grouping of HCW management methods according to the type of technology used. Chemical strategies such as disinfection or immobilization are effective in certain contexts but may generate residues, add volume, or fail to neutralize hazardous agents. Biological enzymatic converters provide low-odor, on-site treatment but are restricted in scope and unsuitable for cytotoxic or anatomical materials. Radiation-based methods (i.e., ultraviolet, gamma, or electron beam) achieve non-thermal pathogen inactivation without chemical residues, though shielding is required and waste volume is unchanged. Thermal technologies, both non-combustion and combustion, remain central, offering rapid and reliable decontamination but differing in efficiency, cost, and environmental footprint. Engineered landfills serve as the ultimate containment option for treated non-hazardous waste, while emerging nanotechnology offers targeted removal capacities, though its application is still experimental and accompanied by toxicological uncertainties [177,178,179,180].

## 6. Conclusions

HCWs represent are critical occupational and environmental threats due to their mixed composition and diverse toxic mechanisms. Sharps and infectious fractions transmit bloodborne pathogens, driving risks of hepatitis and HIV. Pharmaceutical and cytotoxic residues interfere with metabolic pathways, inducing mutagenesis, carcinogenesis, and teratogenesis. Mercury and other heavy metals contribute to immune dysfunction, endocrine imbalance, and multi-organ toxicity, while chlorine-based disinfectants and pesticides are linked to respiratory injury, neurotoxicity, and hormonally mediated cancers. Radioactive materials are particularly dangerous through direct DNA interaction and enhanced malignancy risk.

Risk concentrates among clinical staff, waste handlers, and communities near insufficiently controlled landfills or incineration sites. Effective risk management targets prevention, minimization, and strict segregation at source, with treatment methods matched to stream-specific hazards.

In direct response to RQ1, the review delineates the principal hazard-bearing streams (i.e., infectious, sharps, pharmaceutical/cytotoxic, chemical, pathological, and radioactive) and identifies settings that concentrate generation and exposure potential, especially metropolitan general hospitals and laboratories, with context-specific peaks (i.e., maternities in some systems), wide cross-country heterogeneity in generation rates, and COVID-era surges recognized as exceptional spikes rather than baselines; composition profiles further show plastics and paper dominating non-hazardous fractions, underscoring targeted recycling needs. In response to RQ2, the main exposure routes and toxic mechanisms are mapped by stream, from percutaneous transmission of bloodborne viruses to genotoxic, endocrine, metabolic, and radiological pathways. In response to RQ3, risk-proportionate management is specified by typology, linking stream characteristics to prevention, minimization, segregation, and appropriately matched treatment technologies.

At the point of generation, HCW should be segregated into defined streams (i.e., infectious, sharps, pharmaceutical or cytotoxic, chemical, pathological, radioactive, and general) to enable risk-proportionate handling throughout the chain of custody. Prevention and minimization should be prioritized through waste-sparing procurement, controlled reuse of devices designated as reusable following validated decontamination, and targeted recycling of non-hazardous fractions such as plastics, paper, and metals. Treatment pathways should then be aligned with waste typology. Infectious waste is most effectively managed with non-combustion thermal processes, including steam and microwave technologies. Pathological and pharmaceutical or cytotoxic fractions are best addressed with high-temperature approaches, whereas radioactive residues should be immobilized and contained. Consistent hazard labeling and safe-handling procedures for corrosives and volatile disinfectants should be maintained so that occupational and environmental exposures remain within accepted limits. Institutional governance and workforce capability should be consolidated through a dedicated waste management team with routine training and oversight.

Evidence gaps remain in under-reported settings, in understanding the mechanistic effects of complex waste mixtures, and in evaluating the operational and environmental performance of emerging thermal and radiation technologies, warranting targeted primary studies.

## Figures and Tables

**Table 1:** Classification, description, and associated risks of HCW types.

Type of HCW	Examples	Description	Risks Associated	Refs.
Infectious	Wastewater and materials contaminated with blood or bodily fluids, including laboratory cultures, microbiological stocks, and items from isolation wards used for highly infectious patients	This category includes materials that may facilitate the spread of infectious microorganisms, such as cultures of pathogens and waste from quarantined patients with contagious diseases	Potential to contain pathogenic microorganisms capable of transmitting diseases to humans upon exposure	[27,31]
Pathological	Human remains such as tissues, organs, and fluids, as well as contaminated animal carcasses, fetal tissue, and unused blood products	Organic waste that contains bodily fluids, excreta, or blood, which can contribute to contamination if not managed correctly	May carry infectious agents that could spread diseases, particularly when sourced from infected individuals or animals. Inadequate disposal can lead to environmental contamination, affecting soil and water quality	[32]
Sharps waste	Needles, syringes, scalpels, infusion sets, pipettes, blades, broken glass, and other sharp medical instruments	There is limited research on the disposal of sharps in non-clinical environments, and incorrect disposal increases the likelihood of needle-stick injuries	Sharp objects can puncture or lacerate the skin, allowing direct exposure to infectious agents. Used needles and syringes frequently harbor bloodborne pathogens such as Human Immunodeficiency Virus (HIV), Hepatitis B, and Hepatitis C	[33]
Pharmaceutical and cytotoxic waste	Expired or unused medications, drug-contaminated materials, and cytotoxic waste containing substances with genotoxic properties, including chemotherapy drugs	Cytotoxic waste remains hazardous even after disposal due to its potential to cause genetic mutations, fetal abnormalities, or cancer. It includes chemotherapy agents, genotoxic chemicals, and excreta from patients undergoing cytostatic drug therapy	Improper elimination, such as flushing drugs into water systems, can contaminate natural water bodies, impacting aquatic ecosystems and human water supplies. Incorrect disposal of antibiotics contributes to antimicrobial resistance, making bacterial infections harder to treat	[1,34]
Chemical waste	Laboratory solvents, reagents, disinfectants, sterilants, and heavy metals from medical equipment (e.g., mercury in broken thermometers or discarded batteries)	Hazardous chemical waste exhibits toxic, flammable, corrosive, reactive, or oxidizing properties	Exposure to toxic chemicals can result in poisoning, particularly through inhalation, ingestion, or skin absorption. Strong acids and bases can cause severe tissue damage upon contact, leading to chemical burns or respiratory complications	[1,35]
Radioactive waste	Medical products contaminated with radionuclides, such as diagnostic radiopharmaceuticals and therapeutic radioactive materials	Waste containing radioactive substances that can emit ionizing radiation, requiring specialized disposal and containment methods	Exposure to radioactive materials poses health hazards, including radiation-induced illnesses, DNA mutations, and increased cancer risk. Proper disposal is essential to mitigate environmental and biological contamination	[36]

HCW, healthcare waste; Refs., references.

**Table 2:** Various types of HCW-generating sources.

Large Sources		Medium Sources	Minor Sources
Hospitals	University	Mortuary facilities	Primary Healthcare Physicians
	General		
	District		
Urgent Care and Trauma Units		Palliative Centers	Dental clinics
Maternal Health and Birthing Centers		Agricultural and Equine Veterinary Clinics	Traditional Needle Therapy Practitioners
Dialysis centers		Animal hospitals	Spinal Adjustment and Musculoskeletal Therapy Experts
Blood Processing and Donation Units		Ambulatory Healthcare Facilities	Convalescent nursing homes
Military medical services		-	Mental Health and Behavioral Therapy Institutions
Medical research centers			Disabled persons’ institutions
Advanced Biotech Research Labs			Pharmacies
Animal research and testing			Aesthetic Body Modification Studios
Senior Assisted Living Centers			Residential Medical Assistance Services
Hematology Storage and Donation Centers			Ambulance services

**Table 3:** Overall HCW generation and infectious waste production in different countries.

Country	Overall HCW Generation (kg/bed/day)	Infectious Waste Generation (kg/bed/day)	Healthcare Institution	Refs.
Pakistan	6.762	3.292	Hospitals Clinics and dispensaries Basic health units Consulting clinics Nursing homes Maternity	[54,60,61]
Tanzania	0.21	0.117	Health centers (urban) Rural dispensaries Urban dispensaries	[54,62]
South Africa	–	5.13	Private community health center District hospital Provincial tertiary hospital Public community health center Private day-surgery clinic National central hospital Specialized hospital Regional hospital Public clinic	[54,63]
United States	10.7 (metropolitan general hospitals), 7.7 (laboratories), 0.65 (veterinary, individual metropolitan), 0.93 (rural doctor’s office), 0.043 (psychiatric hospitals), 0.038 (nursing homes)	2.79 (metropolitan general hospitals), 2.03 (rural general hospitals)	Dentist’s office (individual) Veterinary (rural) Doctor’s office (group practice, urban) Metropolitan general hospitals Doctor’s office (rural) Dentist’s office (rural) Nursing homes Veterinary (individual, metropolitan) Public community health center Private community health center Private day-surgery clinic Rural general hospitals Doctor’s office (individual, urban) Psychiatric and other hospitals Regional hospital Provincial tertiary hospital Specialized hospital District hospital Laboratories Public clinic	[1,54,64]
Canada	8.2	–	Hospitals	[57,64]
Spain	3.5–4.4	–	University Hospitals and Regional Hospitals	[6,65,66]
France	0.3–3.6	–	Public and Private Hospitals	[6,13,65]
China	0.6–4.03	–	Large Urban Hospitals	[65,67,68]
India	0.8–2.31	–	Public and Private Hospitals	[13,65,69]
Kazakhstan	5.34–5.4	–	Major Tertiary Hospitals	[7,65,70]
Brazil	2.94–3.3	–	Large University Hospitals and Specialized Care Centers	[6,65,71]
Ethiopia	1.1–1.8	–	District Hospitals and Regional Medical Centers	[16,65,72]
Morocco	0.4–0.7	–	Public Health Centers and Regional Hospitals	[18,65]
Sudan	0.38–0.9	–	District Hospitals and Rural Healthcare Centers	[65,73]

**Table 4:** Associations between exposure to different chemicals and possible diseases.

Chemical	Pathology	Inference Gene Network	Inference Score
Mercury	Diabetes Mellitus, Type 2	AKT2|BAX|BCL2|BCL2L11|BRAF|C3|CASP3|CAT|CYP1A2|ENPP1|FAS|GCLC|GCLM|GPX1|GSTM1|HMOX1|HNF1A|HPX|IL6|INS1|IRS1|LEPR|MIR151A|MIR423|MIRLET7D|NFKB1|NOS2|OGG1|PAX6|SLC2A4|SOD1|TIMP1|TNF|TNFRSF1A|ZFAND3	52.32
	Autism Spectrum Disorder	ABCB1|ABCG2|ACHE|AHR|AKR7A3|ALAD|ALDH5A1|ALDH6A1|AQP4|AQP9|BDNF|CA2|CFTR|CHAT|COMT|CP|CRYZ|CYP1A1|CYP1A2|CYP27A1|CYP2U1|CYP7A1|DRD4|GNGT1|GSTA2|GSTM1|GSTP1|MTR|NOS2|NQO1|RELN|SLC3A2|SLC6A4|TJP1|TXNRD1|TXNRD2	51.93
	Autistic Disorder	ADM|AQP4|BCL2|BDNF|CADM1|CAT|COMT|CP|DAB1|DRD3|EIF4G1|GPX1|GSTM1|GSTP1|HRAS|HTN1|IFNG|IGF1|IL10|IL13|IL2|IL4|IL6|MAPK3|MTF1|NAV3|NOS2|PAX6|PON1|PTGS2|RELN|SLC6A4|TF	49.42
	Chemical and Drug Induced Liver Injury	ABCB1|ABCB1B|ABCC1|ABCC2|ACSL1|ACTB|AHR|ALB|ANXA2|APOE|ARG1|ARNT|BAX|C3|CA3|CAT|CCR2|CP|CPS1|CRP|CYP1A1|CYP1A2|CYP1B1|CYP2A6|EIF2AK1|GCLC|GCLM|GPT|GSN|GSR|GSTA1|GSTM1|GSTM3|GSTP1|GSTT1|HAVCR1|HLADQB1|HMOX1|HPX|HSPA5|IFNG|IGF1|IL1B|IL4|IL6|LCN2|LTF|MIR10A|MIR193B|MIR423|MIRLET7B|MIRLET7C|MIRLET7D|MIRLET7G|MIRLET7I|MMP2|MMP9|NFATC4|NFE2L2|NOX4|PARK7|PON1|PTGS2|SERPING1|SHC1|SLC22A8|SOD1|SPP1|TF|TNF|VIM	46.18
	Obesity	ACHE|ACSL1|AHR|ALDH6A1|APOE|CA3|CASP1|CD40|CRP|CYP1B1|EFNB1|ENPP1|FOS|GAS7|GPX1|GPX3|HMOX1|HSPA5|IL6|IRS1|LEPR|MMP9|NQO1|OGG1|PARP1|PMCH|PTGS2|SLC22A1|SOD1|TF|TNF	42.77
Silver	Diabetes Mellitus, Type 2	ABCC8|ADAMTS9|ATF3|BAX|BCL2|BRAF|CASP3|CAT|CCDC92|CCND2|CYP1A2|EDN1|ENPP1|ETS1|GCG|GCLC|GCLM|GLIS3|GNB3|GPX1|GSTM1|HHEX|HK1|HMOX1|ICAM1|ID1|IL6|IRS2|ITGA1|MIR1226|MIR140|MIR141|MIR142|MIR17HG|MIR181C|MIR192|MIR200A|MIR214|MIR27A|MIR33B|MIR409|MIR483|MIR628|MIR92B|NFKB1|NOS2|NOS3|PAX6|PEPD|PPARG|PROX1|SFRP4|SIRT1|SLC2A4|SNAP25|SOD1|SOD2|ST6GAL1|TCF7L2|TNF	54.74
	Chemical and Drug Induced Liver Injury	AASS|ABCC1|ABCC2|ACTB|ADAM8|AGT|AIFM1|ARG1|BAX|BLVRB|BMAL1|CAT|CCL2|CHRM3|CLU|CP|CTNNB1|CXCL1|CXCL10|CXCL14|CYP1A1|CYP1A2|EIF4EBP2|EPHX1|F3|FGA|FLT1|GADD45A|GCLC|GCLM|GDA|GSR|GSTA4|GSTM1|GSTM2|GSTO1|GSTP1|GSTT1|HADHA|HMOX1|HSPA5|IFNG|IGF1|IL11|IL18|IL1A|IL1B|IL1R2|IL22|IL6|IRAK1|KITL|LDLR|LTF|MALAT1|MAP1LC3B|MDH1|MIR122|MIR1247|MIR1290|MIR132|MIR141|MIR142|MIR150|MIR181C|MIR18A|MIR191|MIR192|MIR19A|MIR200C|MIR22|MIR23A|MIR23B|MIR27B|MIR29B2|MIR33B|MIR362|MIR455|MIR483|MIR484|MIR877|MIRLET7C|MIRLET7G|MMP2|MST1|MTHFR|NFATC4|NFE2L2|NFXL1|NOX4|NR0B2|NR1I2|NR2F2|NREP|NTN1|PARK7|PDK4|PNP|PTGS2|SERPINA6|SESN2|SOD1|SOD2|SOD3|SORD|SPP1|STING1|TBXA2R|TF|THBS1|TNF|TTR|UNC93B1|VEGFA|VIM	20.88
	Nerve Degeneration	ANGPT1|APLP2|APP|ATRN|BAX|BCL2|BDNF|CASP3|CDH1|CDK5R1|CNR1|CP|CTNNB1|DDIT4|EPOR|FGF2|IFNG|IGF1|MAPK1|MAPK3|MT1|MT2|NOS1|OTX2|PANK1|PARK7|PPARG|PSEN1|SELENOP|SIRT1|SLC18A2|SNCA|SOD1|SOD2|TFEB|TNF|XDH	11.05
	Poisoning	GCG|GSK3B	10.4
	Cardiomegaly	AGT|APLN|BAMBI|CCND2|CEBPB|CYP1A1|EDN1|FGF2|FHL2|GSK3B|HMOX1|IGF1|IL18|IL1B|MT2A|NOS3|NPPB|PRKCZ|REN|RRAD|SLC2A4|SOD2|SOX4|TNF|TRPC1	9.48
Chlorine	Myocardial Ischemia	ADRB2|ATP1A1|CCL2|CCL3|CCND1|CPT1B|CRK|CXCL10|EDN1|FABP5|FGF2|GHR|GSTM2|HMGCS2|HMOX1|ICAM1|IGFBP3|IL1A|IL1B|IL6|KCNJ8|MEOX2|NFKBIA|NOS3|NR4A1|PPP2CA|SELE|SELP|TFRC|TNF|TUBA1A	59.7
	Inflammation	AGER|AKT1|APOA1|ATP7B|BDKRB2|CCL11|CCL2|CCL3|CHRNA4|CRHR2|CSF2|CXCL8|EDN1|EGR1|FGF2|HMOX1|ICAM1|IFNG|IL13|IL1A|IL1B|IL6|MMP9|MPO|NOS2|PTGS2|TFRC|TIMP1|TNF|TRPA1	34.3
	Pulmonary Fibrosis	CAT|CCL11|CCL2|CCL3|CCL5|CSF2|CSF3|CXCL8|EDN1|FGF2|HMOX1|IL12B|IL13|IL1B|IL6|MECP2|MMP9|TIMP1|TNF	33.8
	Acute Lung Injury	ACVR1|APOA1|CASP3|CAT|EDN1|FAS|ICAM1|IL1B|IL1RL1|IL6|SFTPD|TNF	20.24
	Burns, Chemical	ALOX12|FGFR2|IL1A|IL1B|IL1R1|IL6	19.03
Aldrin	Prostatic Neoplasms	AR|CDH1|COL15A1|CTSB|CYP19A1|CYP3A4|CYP7B1|ERBB3|ESR1|ESR2|HMOX1|IVNS1ABP|NGFR|NOS3|SPON2|TET2	18.53
	Liver Neoplasms, Experimental	CAR3|CDH1|ESR1|NR1I3|TSC22D1	5.3
	Diabetes Mellitus	NCF1|NR1I2|NR1I3	4.94
Mirex	Skin Neoplasms	CSF3|ODC1	5.41
	Chemical and Drug Induced Liver Injury	ADIPOQ	3.57
Endrin	Adenocarcinoma	CEBPB|CYP26A1|ESR1|ESR2|NFKB1|NR1I2|PGR|PPARG|RARB|TRP53	26.87
	Obesity	ADIPOQ|CEBPA|CYCS|ESR1|FASN|NR1I2|NR1I3|NTRK2|PFKFB3|PPARG	24.94
	Breast Neoplasms	ADAMTS1|ALK|AR|CYP3A4|ESR1|ESR2|FASN|HSP90AA1|KIT|PGR|RARB|TRP53	20.66

Note: The inference score represents a Comparative Toxicogenomics Database-derived metric quantifying the strength of chemical–gene–disease associations (higher scores indicate stronger inferred relationships), based on data curated in the Comparative Toxicogenomics Database [143].

**Table 5:** Toxic mechanisms of HCW exposure.

HCW	Mode of Exposure	Toxic Mechanism	Refs.
Bloodborne Pathogens (HIV, HBV, HCV)	Percutaneous Injuries (sharps, needles, scalpels) contaminated with blood from infected individuals	HIV enters the bloodstream, it binds to CD4 receptors on helper T-cells, fusing with the cell membrane, causing immunodeficiency and making individuals susceptible to opportunistic infections, cancers, and eventually leading to AIDS HBV infects hepatocytes and uses the host’s cellular machinery to replicate HCV targets the liver, leading to chronic infection and inflammation Both HBV and HCV can lead to fibrosis, cirrhosis, and hepatocellular carcinoma	[158,159]
Pharmaceutical and Genotoxic Waste (chemotherapy agents, cytotoxic drugs, expired pharmaceuticals)	Inhalation, dermal absorption, ingestion, or accidental puncture	Drugs like antibiotics can cause acute toxicity by disrupting metabolic processes Cytotoxic drugs interfere with DNA replication and cell division by targeting rapidly dividing cells. Alkylating agents add alkyl groups to DNA, leading to crosslinking and breakage, which results in mutagenesis, carcinogenesis, and teratogenesis. Acute toxicity causes liver, kidney, and gastrointestinal impairment, while prolonged exposure increases the risk of cancer, reproductive damage, and organ dysfunction.	[160,161,162,163]
Mercury	Inhalation (mercury vapor), ingestion, or dermal absorption	Mercury vapor is absorbed through the lungs, entering the bloodstream. It accumulates in organs, particularly the brain, kidneys, and liver. Mercury is neurotoxic and impairs neuronal function by binding to sulfur-containing groups on enzymes, disrupting neurotransmitter release and leading to cognitive dysfunction, tremors, visual and auditory impairments, and kidney damage	[164,165]
Silver	Inhalation of silver dust or fumes, ingestion of contaminated water or food, or dermal absorption from medical applications	Silver accumulates in tissues, particularly the skin, liver, kidneys, and nervous system. Silver ions bind to thiol (-SH) groups in proteins, disrupting enzymatic activity and cellular functions. Silver nanoparticles induce oxidative stress, leading to cellular damage, inflammation, and apoptosis. Argyria causes irreversible skin discoloration, while silver exposure leads to liver and kidney dysfunction, neurotoxicity, cognitive impairment, peripheral neuropathy, and long-term risks of metabolic disorders, immune suppression, and organ toxicity.	[141]
Chlorine	Inhalation, skin contact, or accidental ingestion	When chlorine interacts with water or organic matter, it forms chlorine gas, which can be inhaled. Chlorine gas is a potent respiratory irritant, causing bronchospasm, pulmonary edema, and airway inflammation. It also damages the mucous membranes of the eyes, skin, and respiratory tract by reacting with cellular proteins and lipids. It may cause respiratory distress, lung damage, and ocular irritation.	[166]
Pesticides (Aldrin, mirex, DDT)	Dermal contact, inhalation, or ingestion	Pesticides disrupt endocrine signaling by mimicking or blocking natural hormones. They can induce oxidative stress, which damages cellular components such as lipids, proteins, and DNA. Aldrin, Mirex, and DDT are known to cause cancers, endocrine disruptions and neurotoxicity, leading to tremors, convulsions	[167,168]
Formaldehyde	Inhalation, skin absorption, or ingestion	Causes DNA cross-linking and impairs protein synthesis, leading to cell death. It is a known carcinogen, particularly for nasopharyngeal and lung cancer and may also cause respiratory distress	[169]
Radioactive waste	Emission of gamma radiations, beta radiations and low-energy X-rays	The radiations can interact with DNA, causing mutations that may lead to cancer	[170]

HIV, human immunodeficiency virus; HBV, hepatitis B virus; HCV, hepatitis C virus; AIDS, acquired immunodeficiency syndrome; DNA, deoxyribonucleic acid.

## Data Availability

No new data were created or analyzed in this study.

## Abbreviations

The following abbreviations are used in this manuscript:

HCW	Healthcare Waste
WHO	Directory of open access journals
HIV	Human Immunodeficiency Virus
HCV	Hepatitis C Virus
HBV	Hepatitis B Virus
MRSA	Methicillin-resistant *Staphylococcus aureus*
DNA	Deoxyribonucleic Acid
UV	Ultraviolet
